# Therapy for pneumonitis and sialadenitis by accumulation of CCR2-expressing CD4^+^CD25^+ ^regulatory T cells in MRL/lpr mice

**DOI:** 10.1186/ar2122

**Published:** 2007-02-07

**Authors:** Hitoshi Hasegawa, Atsushi Inoue, Masatake Muraoka, Jun Yamanouchi, Tatsuhiko Miyazaki, Masaki Yasukawa

**Affiliations:** 1 First Department of Internal Medicine, Division of Pathogenomics, Ehime University School of Medicine, Shitsukawa 454, Toon City, Ehime 791-0295, Japan; 2 Department of Pathology, Division of Pathogenomics, Ehime University School of Medicine, Shitsukawa 454, Toon City, Ehime 791-0295, Japan

## Abstract

Adoptive transfer of CD4^+^CD25^+ ^regulatory T cells has been shown to have therapeutic effects in animal models of autoimmune diseases. Chemokines play an important role in the development of autoimmune diseases in animal models and humans. The present study was performed to investigate whether the progression of organ-specific autoimmune diseases could be reduced more markedly by accumulating chemokine receptor-expressing CD4^+^CD25^+ ^regulatory T cells efficiently in target organs in MRL/MpJ-*lpr*/*lpr *(MRL/lpr) mice. CD4^+^CD25^+^Foxp3^+ ^T cells (Treg cells) and CD4^+^CD25^+^Foxp3^+ ^CCR2-transfected T cells (CCR2-Treg cells) were transferred via retro-orbital injection into 12-week-old MRL/lpr mice at the early stage of pneumonitis and sialadenitis, and the pathological changes were evaluated. Expression of monocyte chemoattractant protein-1 (MCP-1)/CCL2 was observed in the lung and submandibular gland of the mice and increased age-dependently. The level of CCR2 expression and MCP-1 chemotactic activity of CCR2-Treg cells were much higher than those of Treg cells. MRL/lpr mice to which CCR2-Treg cells had been transferred showed significantly reduced progression of pneumonitis and sialadenitis in comparison with MRL/lpr mice that had received Treg cells. This was due to more pronounced migration of CCR2-Treg cells and their localization for a longer time in MCP-1-expressing lung and submandibular gland, resulting in stronger suppressive activity. We prepared chemokine receptor-expressing Treg cells and demonstrated their ability to ameliorate disease progression by accumulating in target organs. This method may provide a new therapeutic approach for organ-specific autoimmune diseases in which the target antigens remain undefined.

## Introduction

CD4^+^CD25^+ ^regulatory T cells are critical for the regulation of host tolerance and are considered to have enormous potential for suppressing pathological immune responses in autoimmune disease, transplantation, and graft-versus-host disease (GVHD) [1,2]. These cells express a specific transcription factor, forkhead box p3 (Foxp3), which has been associated with their development and suppressive function [3-5]. The mechanism by which CD4^+^CD25^+ ^regulatory T cells suppress immune responses is poorly understood, but these cells appear to suppress a variety of reactions, including CD4^+ ^and CD8^+ ^T-cell responses as well as B-cell responses [6,7]. CD4^+^CD25^+ ^regulatory T cells have also been reported to act directly on natural killer (NK), natural killer T (NKT), and dendritic cells [8-10]. Therefore, the suppressive characteristics of CD4^+^CD25^+ ^regulatory T cells have made them an attractive candidate for immunotherapy, and numerous studies using animal models have demonstrated their potential for the control of autoimmune diseases as well as transplant rejection and GVHD.

CD4^+^CD25^+ ^regulatory T cells account for only 5% to 10% of the total CD4^+ ^population in both mice and humans [1,2]. Therefore, administration of sufficient numbers of freshly isolated CD4^+^CD25^+ ^regulatory T cells is not therapeutically practical, and it is critically important to expand the number of CD4^+^CD25^+ ^regulatory T cells to facilitate their accumulation in target organs for clinical immunotherapy. Recent studies have shown that suppressive properties and organ-specific tolerance are critically dependent on the antigen specificity of CD4^+^CD25^+ ^regulatory T cells and that antigen-specific CD4^+^CD25^+ ^regulatory T cells are more effective for disease protection than polyclonal CD4^+^CD25^+ ^regulatory T cells, which means that lower numbers of CD4^+^CD25^+ ^regulatory T cells are needed for therapy [11-16]. Thus, it seems likely that therapy using antigen-specific CD4^+^CD25^+ ^regulatory T cells has the most potential for treatment of autoimmune diseases in which target antigens have been identified. However, in the majority of autoimmune diseases in humans, relevant organ-specific antigens remain undefined. Accordingly, we noticed that the chemokine-receptor system accumulates CD4^+^CD25^+ ^regulatory T cells efficiently in target organs.

Chemokines play an important role in the pathogenesis of autoimmune diseases in murine models and humans [17,18]. The MRL/MpJ-*lpr*/*lpr *(MRL/lpr) mouse strain spontaneously develops a severe generalized autoimmune disease resembling systemic lupus erythematosus [19], characterized by sialadenitis, interstitial pneumonitis, lupus nephritis, vasculitis, arthritis, and lymphadenopathy. The major immunopathological phenomenon observed in sialadenitis and pneumonitis is infiltration of mononuclear cells, especially CD4^+ ^T cells. We and others have shown that local generation of chemokines and the presence of chemokine receptors on the infiltrating cells play a critical role in the initiation and progression of autoimmune disease in MRL/lpr mice [20-27]. Monocyte chemoattractant protein-1 (MCP-1)/CCL2 and interferon-inducible protein-10 (IP-10)/CXCL10 have been reported to be involved in the process of sialadenitis and pneumonitis in MRL/lpr mice [22-24,26,27].

In the present study, we prepared CD4^+^CD25^+ ^regulatory T cells in which the receptor for MCP-1, CCR2, was highly expressed. Then, we characterized the CCR2-expressing CD4^+^CD25^+ ^regulatory T cells and their therapeutic effect on sialadenitis and pneumonitis by accumulating them in target organs in MRL/lpr mice.

## Materials and methods

### Mice and cells

MRL/lpr mice (H-2^k^) were purchased from The Jackson Laboratory (Bar Harbor, ME, USA)). MRL/Mp-+/+ (MRL/+) mice (H-2^k^) and DBA/1 mice (H-2^q^) were purchased from Japan SLC, Inc. (Shizuoka, Japan). This study was approved by the animal ethics committee of Ehime University (Ehime, Japan). The packaging cell line, PLAT-E, was kindly provided by Prof. Toshio Kitamura (Institute of Medical Science, University of Tokyo, Tokyo, Japan). PLAT-E cells were maintained in Dulbecco's modified Eagle's medium supplemented with 10% heat-inactivated fetal calf serum (FCS) (Life Technologies, Inc., now part of Invitrogen Corporation, Carlsbad, CA, USA).

### Antibodies and reagents

Biotin-conjugated anti-mouse MCP-1 goat polyclonal antibody was obtained from R&D Systems, Inc. (Minneapolis, MN, USA). The hamster anti-mouse CD3e rat monoclonal antibody (MoAb) (145-2C11) was purchased from BD Biosciences (San Jose, CA, USA). Mouse MCP-1 and interleukin-2 (IL-2) were obtained from R&D Systems, Inc.

### *In vitro *expansion of CD4^+^CD25^+ ^T cells

The CD4^+^CD25^+ ^T cells were purified from spleen cells of MRL/+ mice using a CD4^+^CD25^+ ^regulatory T-cell isolation kit (Miltenyi Biotec, Bergisch Gladbach, Germany) according to the manufacturer's recommendation. The purity of the CD4^+^CD25^+ ^T cells was ascertained by flow cytometry analysis using specific antibodies labeled with phycoerythrin or fluorescein isothiocyanate and was found to be more than 95%.

One hundred thousand CD4^+^CD25^+ ^T cells were plated in 24-well flat-bottom plates coated with 5 μg/ml anti-mouse CD3e MoAb in a final volume of 2 ml of complete RPMI medium (RPMI-1640 medium supplemented with 10% FCS, 2 mM l-glutamine, 2 × 10^-5 ^M 2-mercaptoethanol, 100 IU/ml penicillin, and 100 μg/ml streptomycin) in the presence of 2 μg/ml anti-mouse CD3e MoAb and 20 ng/ml IL-2 with 4 × 10^5 ^irradiated (15 Gy) syngeneic splenocytes as feeder cells that had been depleted of T cells by negative selection using a mouse Pan T-cell isolation kit (Miltenyi Biotec) according to the manufacturer's protocol. The medium was changed, anti-mouse CD3e MoAb and IL-2 were added every 3 to 4 days, and fresh feeder cells were added every 10 to 14 days.

### Construction of mouse *CCR2 *or *Foxp3 *gene expression retroviral vectors and production of recombinant retrovirus

The pMXs-IG and pMXs-IR retroviral vectors [28] were kindly provided by Prof. T Kitamura. The pMXs-IG and pMXs-IR retroviral vectors contain a green fluorescent protein and a red fluorescent protein, respectively. The pMXs-IG-mCCR2 vector, in which the fragment containing the coding region of mouse *CCR2 *gene was inserted into the SalI-NotI site of the pMXs-IG vector, was kindly provided by Prof. Osamu Yoshie (Kinki University School of Medicine, Osaka, Japan). The fragment containing the coding region of the mouse *Foxp3 *gene was inserted into the EcoRI-NotI site of the pMXs-IR vector.

The recombinant retrovirus was produced as described by Kitamura and colleagues [29]. Briefly, 2 × 10^6 ^PLAT-E cells were seeded onto 60-mm dishes 1 day before transfection using FuGENE 6 transfection reagent (Roche Diagnostics, Indianapolis, IN, USA) according to the manufacturer's protocol. Cells were cultured for 48 hours and then the retroviral supernatant was stocked for infection of target cells. The estimated titers of the retroviruses were 1 × 10^6 ^to 3 × 10^6 ^infectious units per milliliter based on the infected NIH3T3 cells.

### Infection and expression of recombinant retroviruses

Infection and expression of recombinant retroviruses into CD4^+^CD25^+ ^T cells were performed as described by Kitamura and colleagues [29]. First, we prepared CD4^+^CD25^+^Foxp3^+ ^T cells (Treg cells) by infection of recombinant retrovirus carrying the pMXs-IR-Foxp3 vector into CD4^+^CD25^+ ^T cells. One million cultured CD4^+^CD25^+ ^T cells were suspended with virus stock that had been adjusted to a multiplicity of infection of 5 and then were centrifuged at 1,000*g *for 2 hours at 32°C. These cells then were grown in complete RPMI medium in the presence of anti-CD3e MoAb and IL-2 with irradiated syngeneic splenocytes. After 2 days, Treg cells were isolated by sorting the highly expressed population. Next, CD4^+^CD25^+^Foxp3^+ ^CCR2-transfected T cells (CCR2-Treg cells) were isolated by fluorescence-activated cell sorting after infection with recombinant retrovirus carrying the pMXs-IG-mCCR2 vector into the above Treg cells as described above.

### Total RNA preparation, cDNA synthesis, and real-time polymerase chain reaction for *MCP-1*, *CCR2*, and *Foxp3*

The expressions of *MCP-1*, *CCR2*, and *Foxp3 *were quantified by real-time polymerase chain reaction (PCR). Briefly, for quantitative real-time PCR (qPCR) analysis, total RNAs were extracted from cultured cells, snap-frozen lungs, and submandibular glands using TRIZOL reagent (Invitrogen Corporation). The cDNA was prepared with a SuperScript^™ ^III CellsDirect cDNA synthesis system (Invitrogen Corporation). The expressions of the *MCP-1*, *CCR2*, and *Foxp3 *genes were quantified using a QuantiTect^™ ^SYBR Green PCR kit (Qiagen Inc., Valencia, CA, USA) in a 7500 Real Time PCR System (Applied Biosystems Japan Ltd., Tokyo, Japan) and were corrected with a hypoxanthine phosphoribosyl transferase 1 (HPRT1) control. Amplifications were carried out in a total volume of 25 μl for 45 cycles of 15 seconds at 95°C and 1 minute at 60°C. Samples were run in triplicate and their relative expression was determined by normalizing the expression of each target to HPRT1 and then comparing this normalized value with the normalized expression in a reference control sample to calculate the fold-change value. The primers for the amplicons spanned intron/exon boundaries to minimize any amplification of genomic DNA. The primer sequences were as follows: *MCP-1*, forward 5'-CCAACTCTCACTGAAGCCAGCTC-3' and reverse 5'-TTGGGATCATCTTGCTGGTGAA-3'; *CCR2*, forward 5'-TTACCTCAGTTCATCCACGG-3' and reverse 5'-TCATCGTAGTCATACGGTGT-3'; *Foxp3*, forward 5'-CCCAGGAAAGACAGCAACCTT-3' and reverse 5'-TTCTCACAACCAGGCCACTTG-3'; and *HPRT1*, forward 5'-CAGGCCAGACTTTGTTGGAT-3' and reverse 5'-TTGCGCTCATCTTAGGCTTT-3'.

### Adoptive transfer

Cultured Treg cells and CCR2-Treg cells (1 × 10^5 ^or 5 × 10^5 ^cells) were transferred through retro-orbital venous plexus into 12-week-old MRL/lpr mice (at the early stage of sialadenitis and pneumonitis) twice every 2 weeks. To examine the pathological alterations, the mice were sacrificed 3 weeks after the last injection (that is, when the mice were 17 weeks old).

To examine the kinetics of cells remaining in the lungs and submandibular glands, 5 × 10^5 ^Treg cells or CCR2-Treg cells were injected into the retro-orbital vein of 14-week-old MRL/lpr mice. Then, the lungs and submandibular glands were harvested at 3 hours, 24 hours, 72 hours, 5 days, and 7 days after injection.

### Preparation of mononuclear cells from lungs and submandibular glands

Mice were sacrificed at each time point after injection of Treg cells and CCR2-Treg cells. The chest cavity was opened by sterile surgical dissection, and the inferior vena cava and abdominal aorta were clamped. The left atrium was opened by incision, and the right ventricle was infused with sterile phosphate-buffered saline (PBS) to remove any residual blood in the pulmonary vasculature. The right lung and submandibular gland were removed, snap-frozen in liquid nitrogen, and stored at -80°C for extraction of total RNA. The right lung and submandibular gland were cut into small pieces and placed in RPMI-1640 medium containing 5% FCS, 1 mg/ml collagenase (Sigma-Aldrich, St. Louis, MO, USA), and 100 units per milliliter DNase (Takara Shuzo Co., Ltd., Kyoto, Japan). After 30 minutes of collagenase digestion in a 37°C water bath, these tissues were further disrupted by aspiration through an 18-gauge needle. The collagenase-digested tissues were then subjected to Ficoll centrifugation. The interface between the medium and the Ficoll was removed and washed twice with RPMI-1640 medium containing 5% FCS. The staining cells were examined on an Olympus IX70 inverted microscope (Olympus, Tokyo, Japan).

### Immunohistochemistry

To detect MCP-1 expression, formalin-fixed sections were deparaffinized and analyzed by the avidin-biotin-peroxidase method, using biotin-labeled goat anti-murine MCP-1 polyclonal antibody as described previously [20]. Preimmune biotin-labeled goat serum served as a negative control.

### Evaluation of sialadenitis and pneumonitis

Tissues were fixed in 10% formalin for 24 hours at 4°C, and then paraffin sections were prepared, stained with hematoxylin and eosin. The degree of sialadenitis in the submandibular glands was categorized as 0, normal; 1, mild (cell infiltration localized in the perivascular and/or periductal regions); 2, moderate (cell infiltration not only localized in the perivascular and/or periductal regions but also extending to the parenchyma); or 3, severe (cell infiltration localized in the perivascular and/or periductal regions, with fibrosis and/or granuloma). The sialadenitis index was indicated as the sum of all the scores per section divided by the sum of all vessels and all ducts per section.

The perivascular and peribronchiolar infiltrates were scored on the basis of histopathological findings: 0, normal; 1, less than three cell layers; 2, three to six cell layers; or 3, more than six layers. The index of perivascular lesion was indicated as the sum of all the scores per section divided by the number of all vessels per section. The index of peribronchiolar lesion was indicated as the sum of all the scores per section divided by the number of all bronchioli per section.

The infiltrates in alveolar areas in high-power fields (× 400 magnification) were scored as follows: 0, no infiltrating mononuclear cells; 1, less than 10 infiltrating cells; 2, less than 20 infiltrating cells; or 3, more than 20 infiltrating cells. The alveolar lesions index was indicated as the mean value of 20 random fields per section. The sections were evaluated by one of us, who was blinded to the treatment given.

### Chemotactic assay

Chemotactic assays for cultured Treg cells and CCR2-Treg cells were performed in polycarbonate-membrane, 6.5-mm diameter, 5-μm-pore-size transwell cell culture chambers (Costar Corp, now part of Corning Life Sciences, Acton, MA, USA) as described previously [30]. Aliquots (100 μl) of cells (5 × 10^6 ^per milliliter) suspended in RPMI-1640/0.5% bovine serum albumin were added to the upper chambers. The MCP-1 was added to the lower wells at various concentrations. The cells were allowed to migrate for 2 hours at 37°C in a 5% CO_2 _incubator, after which the filters were fixed with 1% glutaraldehyde in PBS for 30 minutes and stained with 0.5% toluidine blue overnight. Cell migration was quantified by counting the cells in each lower chamber and cells adhering to the bottom of the polycarbonate filter.

### *In vitro *suppression assay

To test the suppressive action of cultured Treg cells and CCR2-Treg cells on alloantigen stimulation, 1 × 10^5 ^freshly isolated CD3^+ ^T cells from MRL/+ mice were stimulated with 1 × 10^5 ^irradiated (15 Gy) allogeneic splenocytes from DBA1 mice. Graded numbers (1.25 × 10^4^, 2.5 × 10^4^, 5 × 10^4^, or 1 × 10^5^) of the above cultured cells were added to the cultures, and the cells were cocultured in a final volume of 200 μl of complete RPMI medium in 96-well round-bottom plates for 4 days. The wells were pulsed with 1 μCi [^3^H]thymidine (Amersham Biosciences, now part of GE Healthcare, Little Chalfont, Buckinghamshire, UK) 18 hours before harvesting.

### Evaluation of circulating immune complexes and anti-DNA antibody

The levels of circulating immune complexes (ICs) and anti-DNA antibody were measured by enzyme-linked immunosorbent assay (ELISA) as described previously [31]. Briefly, ELISA plates were coated with 10 μg/ml human C1q (Sigma-Aldrich) for measurement of circulating ICs or with 5 μg/ml double-stranded calf thymus DNA (Sigma-Aldrich) for measurement of anti-DNA antibody.

## Results

### MCP-1 expression in lungs and submandibular glands of MRL/lpr mice

MRL/lpr mice demonstrated progressive development of pneumonitis and sialadenitis, which became noticeable at 10 to 12 weeks of age. In pneumonitis, mononuclear cell infiltration became noticeable in the perivascular, peribronchial, and alveolar areas. Lesion severity increased with the number of inflammatory cells in the pulmonary parenchyma. Interstitial fibrosis and alveolar atelectasis were observed in the later phase. In sialadenitis, small focal infiltrates of inflammatory cells (< 50 cells) were located predominantly around the blood vessels. After 12 weeks of age, larger infiltrates of mononuclear cells, especially CD4^+ ^T cells, became apparent in the periductular regions, extending to the parenchyma and resulting in parenchymal destruction.

**Figure 1:**
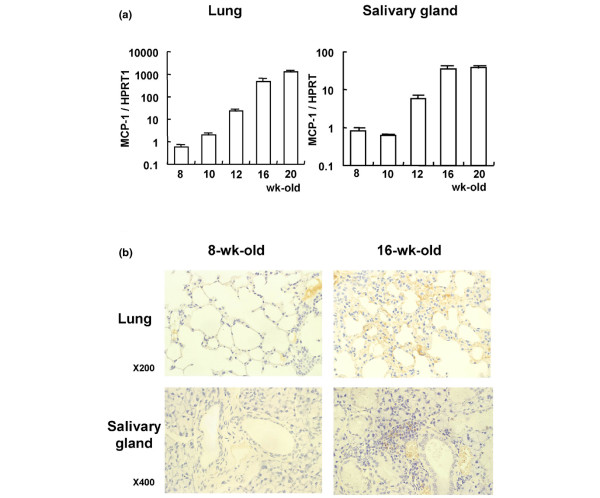
Monocyte chemoattractant protein-1 (MCP-1) expression in the lungs and submandibular glands of MRL/lpr mice. **(a) **Quantitative real-time polymerase chain reaction analysis was performed on total RNA prepared from lungs and submandibular glands of five mice at ages 8, 10, 12, 16, and 20 weeks during the development of pneumonitis and sialadenitis as described in Materials and methods. Results are calculated as a ratio of *MCP-1 *expression to the expression of *hypoxanthine phosphoribosyl transferase 1 (HPRT1)*. **(b) **Representative immunohistochemistry results are shown. Formalin-fixed sections were deparaffinized and incubated with biotin-labeled goat anti-mouse MCP-1 polyclonal antibody, and the expression of MCP-1 was detected with avidin-biotin-peroxidase. The sections were counterstained with hematoxylin. MRL/lpr, MRL/MpJ-*lpr*/*lpr*.

First, we analyzed the expression of *MCP-1 *mRNA by qPCR of total RNA from lungs and submandibular glands during disease development in MRL/lpr mice. As shown in Figure 1a, MRL/lpr mice began to show a significant increase in both lung and submandibular gland *MCP-1 *mRNA at 12 weeks of age and exhibited progressive increases in *MCP-1 *expression from 12 to 20 weeks of age. The *MCP-1 *mRNA levels in lungs of 16- and 20-week-old mice were 20- and 56-fold higher, respectively, than those of 12-week-old mice, whereas the corresponding levels for submandibular glands of 16- and 20-week-old mice were six- and seven-fold higher, respectively, than those of 12-week-old mice. In addition, the level of *MCP-1 *expression in the lung was higher than that in the submandibular gland.

To clarify the localization of MCP-1 expression, immunohistological staining of lung and submandibular gland tissues was performed during disease development (Figure 1b). MCP-1 was expressed significantly in these organs in 16-week-old MRL/lpr mice compared with those in 8-week-old MRL/lpr mice. In the lung, MCP-1 was expressed predominantly in alveolar epithelial cells, macrophages, vascular endothelial cells, and interstitial cells and was also observed occasionally in bronchiolial epithelial cells. In the submandibular gland, MCP-1 was expressed mainly in infiltrating macrophages/monocytes and interstitial cells, but no expression was observed in the ductal epithelium.

### Characterization of Treg cells and CCR2-Treg cells

The CD4^+^CD25^+ ^T cells were separated with microbeads and then cultured with irradiated syngeneic splenocytes in the presence of anti-mouse CD3e MoAb and IL-2. However, after 2 weeks of culture, 10% to 30% of the cells were CD4^+^CD25^+^Foxp3^- ^T cells because contaminating CD4^+^CD25^- ^T cells grew faster than CD4^+^CD25^+ ^regulatory T cells. The cultured CD4^+^CD25^+ ^T cell population, including activated nonregulatory CD25^+ ^T cells such as CD4^+^CD25^+^Foxp3^- ^T cells, is functionally heterogeneous. Therefore, we reprogrammed these cells by ectopic expression of Foxp3 to maintain their suppressive activity. First, we prepared Treg cells by infection of CD4^+^CD25^+ ^T cells with recombinant retrovirus carrying the pMXs-IR-Foxp3 vector. Next, CCR2-Treg cells were isolated after infection of the above Treg cells with recombinant retrovirus carrying the pMXs-IG-mCCR2 vector. There was no significant difference in Foxp3 expression between Treg cells and CCR2-Treg cells, and the two cell types showed similar suppressive activity during at least 1 month (data not shown).

**Figure 2:**
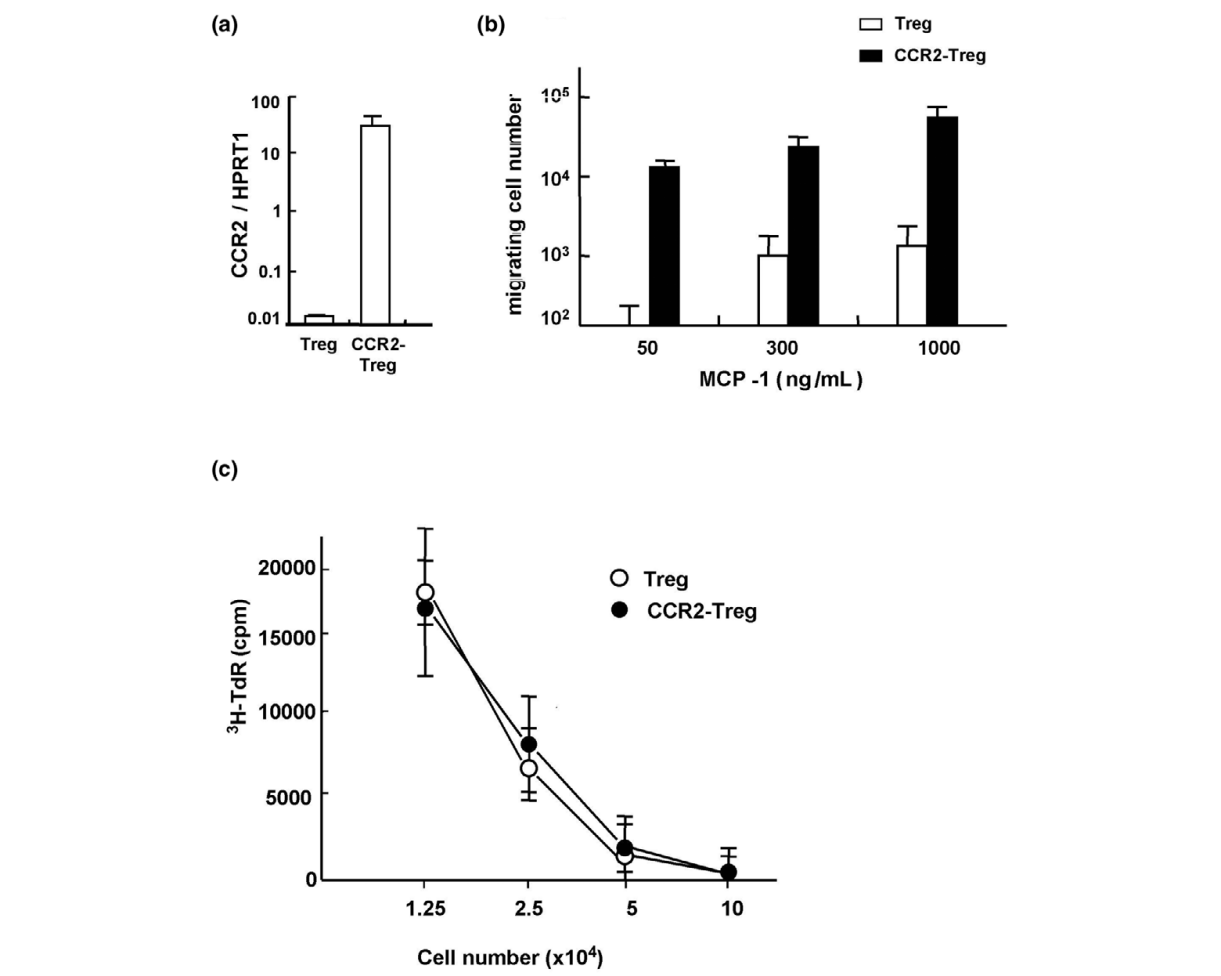
Characterization of Treg cells and CCR2-Treg cells. **(a) **Expression of *CCR2 *gene in Treg cells and CCR2-Treg cells. Quantitative real-time polymerase chain reaction analysis was performed on total RNA prepared from Treg and CCR2-Treg cells. Results are calculated as a ratio of *CCR2 *expression to the expression of *hypoxanthine phosphoribosyl transferase 1 (HPRT1)*. **(b) **Chemotactic activity of Treg and CCR2-Treg cells to monocyte chemoattractant protein-1 (MCP-1). Aliquots (100 μl) of cells (5 × 10^6 ^per milliliter) were added to the upper chambers, and MCP-1 was added to the lower wells at various concentrations. After 2 hours, cell migration was quantified by counting cells in each lower chamber and cells adhering to the bottom part of the filter. Each assay was performed in triplicate. **(c) **Suppressive function of Treg and CCR2-Treg cells to alloantigen. One hundred thousand freshly isolated CD3^+ ^T cells from MRL/+ mice were stimulated with 1 × 10^5 ^irradiated allogeneic splenocytes from DBA1 mice. Graded numbers of the Treg and CCR2-Treg cells were added to the cultures. Wells were pulsed with [^3^H]thymidine (^3^H-TdR) for the last 18 hours of the 4-day culture. Control (no addition of regulatory T cells) is 16,429 ± 5,160 cpm. CCR2-Treg cell, CD4^+^CD25^+^Foxp3^+ ^CCR2-transfected T cell; Treg cell, CD4^+^CD25^+^Foxp3^+ ^T cell.

Expression of the *CCR2 *receptor in cultured Treg cells and CCR2-Treg cells was analyzed by qPCR. As shown in Figure 2a, the expression level of *CCR2 *in CCR2-Treg cells was 2,300-fold higher than that in Treg cells. In the MCP-1-driven chemotactic assay, the chemotactic activity of CCR2-Treg cells was much higher than that of Treg cells (Figure 2b). These findings indicated that CCR2-Treg cells migrated to MCP-1 more efficiently than Treg cells.

The suppressive properties of cultured Treg cells and CCR2-Treg cells were examined in alloantigen-stimulated cultures. As shown in Figure 2c, 2.5 × 10^4 ^cultured Treg cells and CCR2-Treg cells produced a 67% and 57% reduction of the proliferative response, respectively, and the suppressive effects were dose-dependent. There was no significant difference in the suppressive properties between cultured Treg cells and CCR2-Treg cells.

### Evaluation of pneumonitis and sialadenitis in Treg cell- and CCR2-Treg cell-transferred MRL/lpr mice

**Figure 3:**
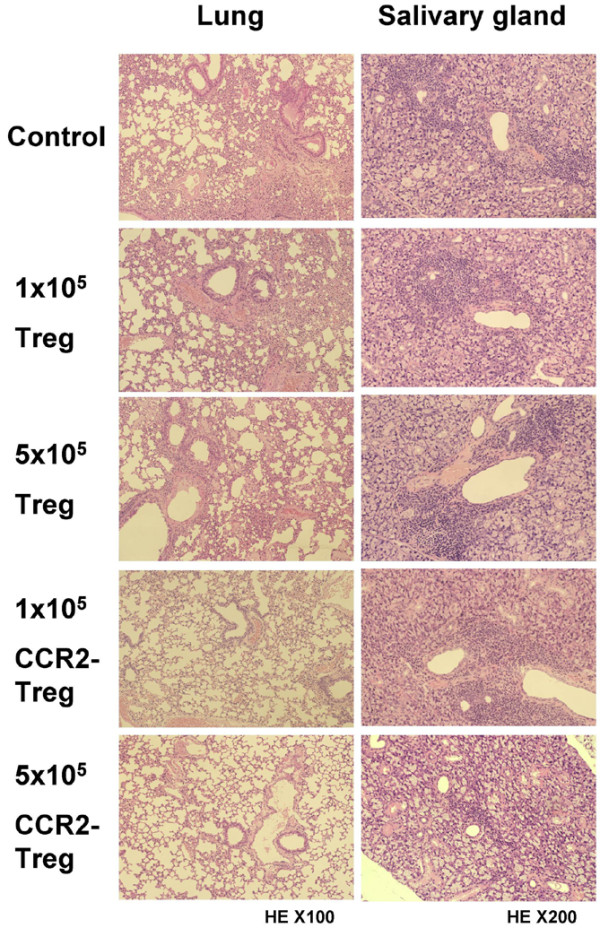
Comparison of pneumonitis and sialadenitis in Treg cell- and CCR2-Treg cell-transferred MRL/lpr mice. Representative photographs are shown. Treg and CCR2-Treg cells were transferred via retro-orbital injection in 12-week-old MRL/lpr mice. One hundred thousand or 5 × 10^5 ^cells were injected into mice twice every 2 weeks, and the pathological changes were evaluated 3 weeks after the last injection (that is, when the mice were 17 weeks old). Non-treated 17-week-old MRL/lpr mice were used as control. CCR2-Treg cell, CD4^+^CD25^+^Foxp3^+ ^CCR2-transfected T cell; HE, hematoxylin and eosin; MRL/lpr, MRL/MpJ-*lpr*/*lpr*; Treg cell, CD4^+^CD25^+^Foxp3^+ ^T cell.

**Figure 4:**
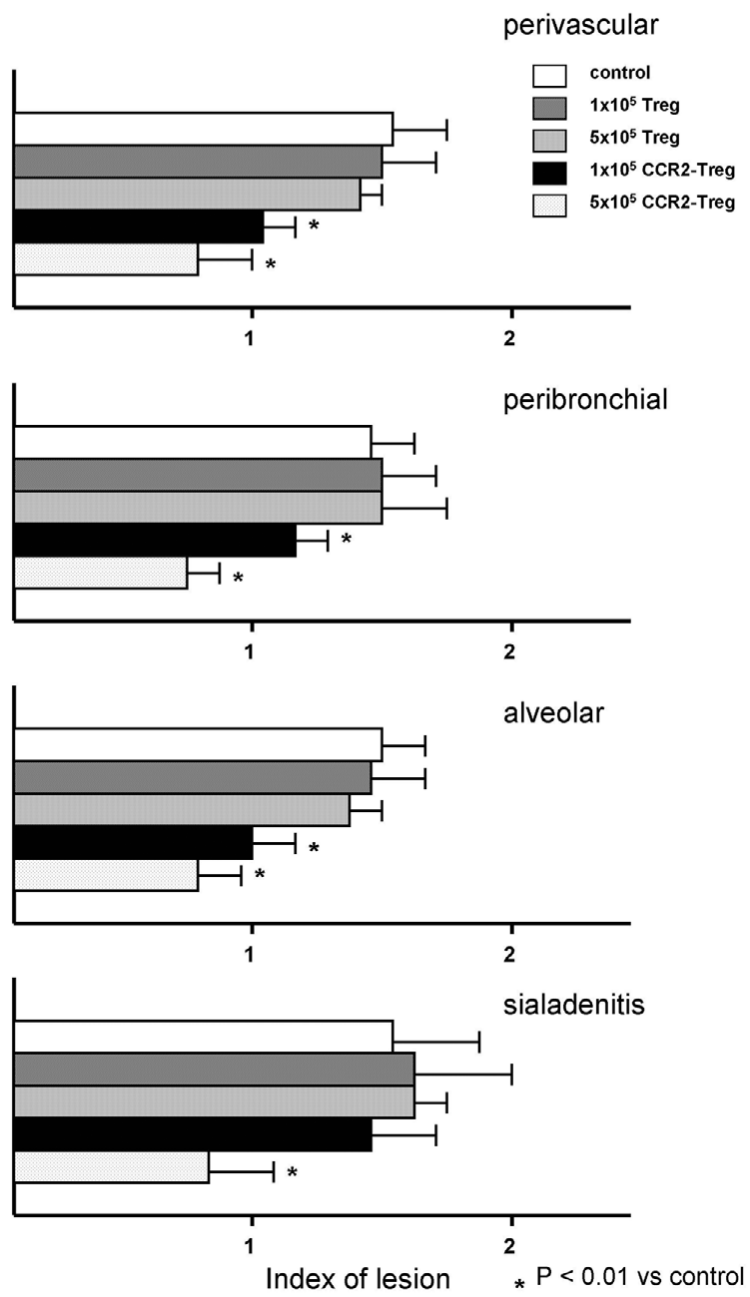
Evaluation of pneumonitis and sialadenitis in Treg cell- and CCR2-Treg cell-transferred MRL/lpr mice. The degrees of pneumonitis and sialadenitis of control and treated 17-week-old MRL/lpr mice were scored as described in Materials and methods. Values are presented as the mean and standard deviation (*n *= 5 to 7 mice per group). Similar results were observed in two independent experiments. *P < 0.01 versus control by Student's *t *test. CCR2-Treg cell, CD4^+^CD25^+^Foxp3^+ ^CCR2-transfected T cell; MRL/lpr, MRL/MpJ-*lpr*/*lpr*; Treg cell, CD4^+^CD25^+^Foxp3^+ ^T cell.

Cultured Treg cells and CCR2-Treg cells were transferred via retro-orbital injection into 12-week-old MRL/lpr mice (at the early stage of pneumonitis and sialadenitis). Either 1 × 10^5 ^or 5 × 10^5 ^cells were injected into the mice twice every 2 weeks, and then the mice were sacrificed 3 weeks after the last injection to evaluate the pathological changes. As shown in Figures 3 and 4, MRL/lpr mice that had undergone transfer of either 1 × 10^5 ^or 5 × 10^5 ^CCR2-Treg cells demonstrated significantly reduced (*P *< 0.01) infiltration of mononuclear cells into the perivascular and peribronchiolar lesions and alveolar areas in comparison with Treg cell-transferred MRL/lpr mice (lesion index: perivascular 1.06 ± 0.14 versus 1.52 ± 0.22 and 0.70 ± 0.19 versus 1.41 ± 0.05, peribronchiolar 1.14 ± 0.10 versus 1.52 ± 0.19 and 0.65 ± 0.13 versus 1.54 ± 0.22, and alveolar 1.02 ± 0.13 versus 1.41 ± 0.20 and 0.71 ± 0.13 versus 1.37 ± 0.11, respectively). On the other hand, there was no significant difference of mononuclear cell infiltration in these areas among control MRL/lpr mice and mice that had undergone transfer of 1 × 10^5 ^or 5 × 10^5 ^Treg cells.

**Figure 5:**
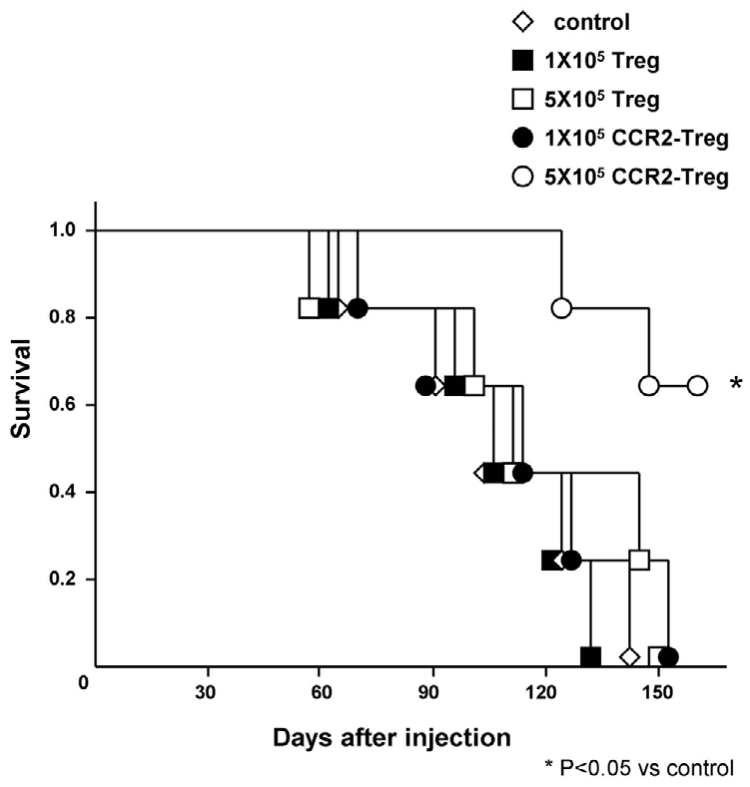
Survival of MRL/lpr mice after transfer of Treg and CCR2-Treg cells. Mice were observed until day 160 or death. All experiments were carried out with groups of five mice. Similar results were obtained in one additional experiment. *P < 0.05 versus control by Student's *t *test. CCR2-Treg cell, CD4^+^CD25^+^Foxp3^+ ^CCR2-transfected T cell; MRL/lpr, MRL/MpJ-*lpr*/*lpr*; Treg cell, CD4^+^CD25^+^Foxp3^+ ^T cell.

In sialadenitis, only MRL/lpr mice that had undergone transfer of 5 × 10^5 ^CCR2-Treg cells showed significantly lower levels of periductal mononuclear cell infiltration and parenchymal destruction compared with the other four groups. No significant difference in the severity of glomerulonephritis or renal vasculitis was observed between Treg cell- and CCR2-Treg cell-transferred MRL/lpr mice that had received either 1 × 10^5 ^or 5 × 10^5 ^cells (data not shown). In addition, there was no significant difference in the production of anti-DNA antibody and circulating ICs among the five groups (data not shown). These findings suggest that CCR2-Treg cells migrate easily into MCP-1-expressing organs and show local rather than systemic suppressive activity in a dose-dependent manner. Furthermore, MRL/lpr mice that had undergone transfer of 5 × 10^5 ^CCR2-Treg cells showed a significant increase in survival compared with the other four groups (Figure 5). This is due to marked reduction of pneumonitis.

In an additional experiment, we prepared CD4^+^CD25^- ^CCR2-transfected T cells by transduction of recombinant retrovirus carrying the pMXs-IG-mCCR2 vector into CD4^+^CD25^- ^T cells and compared pneumonitis and sialadenitis among control, CD4^+^CD25^- ^T cell-transferred, and CD4^+^CD25^- ^CCR2-transferred MRL/lpr mice. Five hundred thousand CD4^+^CD25^- ^T cells and CD4^+^CD25^- ^CCR2-transfected T cells were transferred into 12-week-old MRL/lpr mice as described in Materials and methods. There were no significant differences in pneumonitis and sialadenitis among control, CD4^+^CD25^- ^T cell-transferred, and CD4^+^CD25^- ^CCR2-transferred MRL/lpr mice (lesion index: perivascular 1.48 ± 0.23 versus 1.51 ± 0.07 versus 1.35 ± 0.41, peribronchiolar 1.48 ± 0.41 versus 1.55 ± 0.22 versus 1.49 ± 0.19, alveolar 1.25 ± 0.18 versus 1.29 ± 0.17 versus 1.24 ± 0.11, and sialadenitis 1.24 ± 0.19 versus 1.19 ± 0.37 versus 1.20 ± 0.33, respectively; *n *= 4 per group). Therefore, MRL/lpr mice that had undergone transfer of CCR2-Treg cells showed significant amelioration of pneumonitis and sialadenitis progression in comparison with CD4^+^CD25^- ^CCR2-transferred MRL/lpr mice.

### Localization of adoptively transferred Treg cells and CCR2-Treg cells in the lungs and submandibular glands of MRL/lpr mice

**Figure 6:**
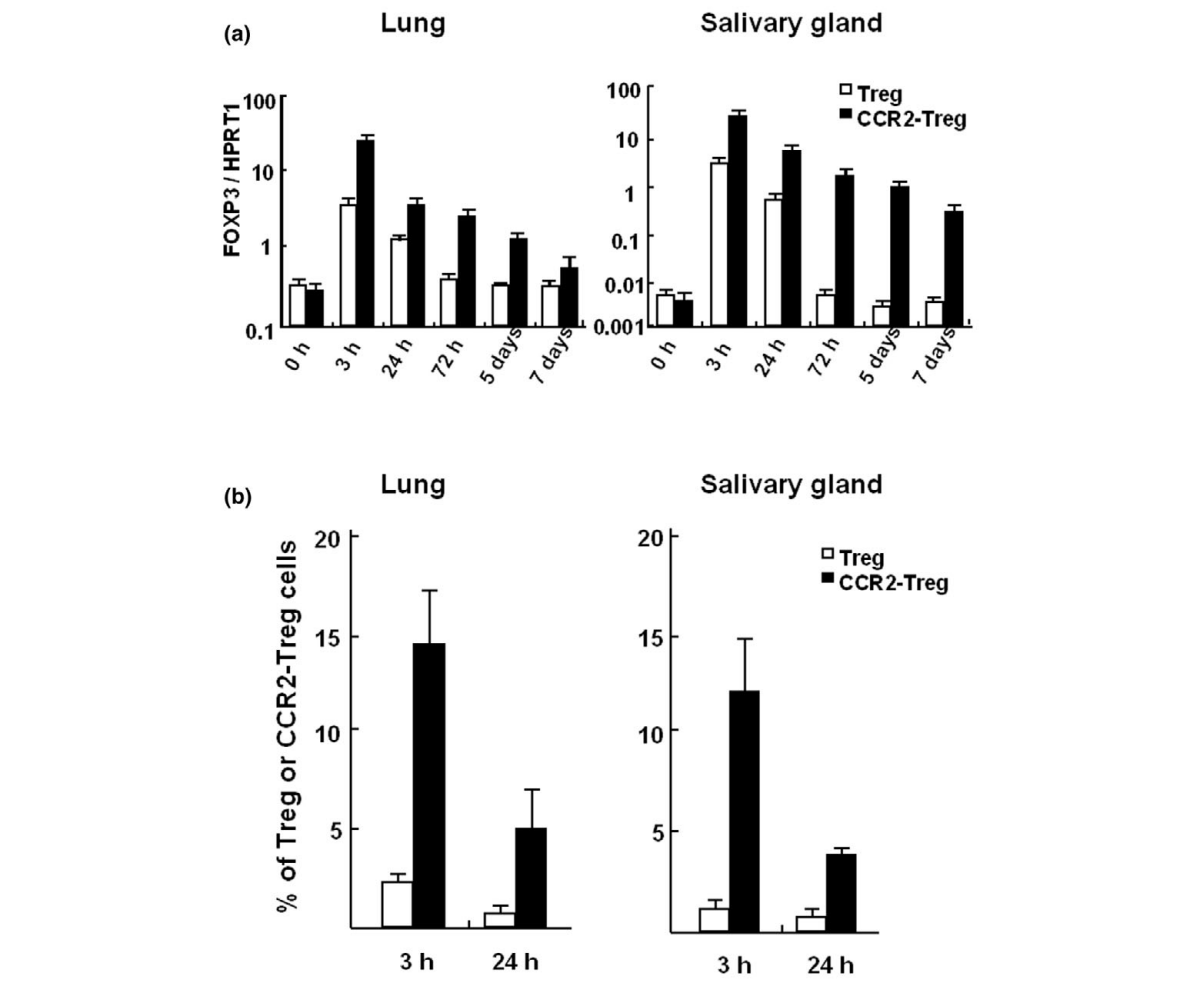
Kinetics of *Foxp3 *expression in the tissues after transfer of Treg and CCR2-Treg cells. **(a) **Five hundred thousand Treg or CCR2-Treg cells were injected into the retro-orbital vein of 14-week-old MRL/lpr mice. Then, the lungs and submandibular glands were harvested at 3 hours, 24 hours, 72 hours, 5 days, and 7 days after transfer. Quantitative real-time polymerase chain reaction analysis was performed on total RNA prepared from the above tissues. Results are calculated as a ratio of *Foxp3 *expression to the expression of *hypoxanthine phosphoribosyl transferase 1 (HPRT1)*. **(b) **Analysis of the mononuclear cells in the lungs and submandibular glands of MRL/lpr mice after transfer of Treg and CCR2-Treg cells. The mononuclear cells in the lungs and submandibular glands were harvested at 3 hours and 24 hours after transfer as described in Materials and methods and analyzed by immunofluorescence on an Olympus IX70 inverted microscope. CCR2-Treg cells contain green fluorescent protein and red fluorescent protein (DsRed), whereas Treg cells contain only DsRed. Results are shown as a percentage of staining cells (Treg or CCR2-Treg cells) (*n *= 3 or 4 mice per group). CCR2-Treg cell, CD4^+^CD25^+^Foxp3^+ ^CCR2-transfected T cell; Foxp3, forkhead box p3; MRL/lpr, MRL/MpJ-*lpr*/*lpr*; Treg cell, CD4^+^CD25^+^Foxp3^+ ^T cell.

Next, to compare the migration of Treg cells and CCR2-Treg cells in the lung and submandibular gland, we harvested these organs at various times after retro-orbital injection in 14-week-old MRL/lpr mice, examined the *Foxp3 *expression in each group by qPCR, and analyzed the mononuclear cells isolated from the lungs and submandibular glands in each group by immunofluorescence. As shown in Figure 6, the localization of CCR2-Treg cells to the lungs was increased more than five-fold both at 3 hours and at 24 hours after transfer compared with that of Treg cells, and the regional localization continued to increase until 7 days. The kinetics of cell localization in the submandibular glands showed a pattern similar to those in the lungs. On the other hand, the numbers of Treg and CCR2-Treg cells localized in the kidneys were significantly lower (Foxp3/HPRT1 ratio at 3 hours: 0.56 ± 0.10 versus 1.94 ± 0.24, respectively) than those in the lungs and submandibular glands, even though MCP-1 was expressed in the kidney of 14-week-old MRL/lpr mice. These findings indicate that, in comparison with Treg cells, more CCR2-Treg cells migrate into MCP-1-expressing lung and submandibular gland and remain localized there for a longer time, resulting in stronger suppressive activity.

## Discussion

In the present study using a murine model, we demonstrated the potential of a new therapeutic approach for markedly reducing the progression of organ-specific autoimmune diseases through the efficient accumulation of chemokine receptor-expressing CD4^+^CD25^+ ^regulatory T cells in target organs. Our main findings were as follows: (a) MCP-1 expression was observed in the lung and submandibular gland of 12-week-old MRL/lpr mice and increased with age, (b) Treg and CCR2-Treg cells had almost the same suppressive properties, (c) MCP-1-directed chemotactic activity of CCR2-Treg was much higher than that of Treg cells, (d) MRL/lpr mice that had undergone transfer of CCR2-Treg cells showed significant amelioration of the progression of pneumonitis and sialadenitis in comparison with Treg cell-transferred MRL/lpr mice, and (e) this effect was due to the migration of higher numbers of CCR2-Treg cells into MCP-1-expressing lung and submandibular gland and their localization there for a longer time, resulting in stronger suppressive activity.

Naturally occurring CD4^+^CD25^+ ^regulatory T cells, which show high expression of Foxp3, are essential for the maintenance of peripheral tolerance through their suppression of autoreactive T cells [1,2]. CD4^+^CD25^+ ^regulatory T cells are able not only to suppress CD4^+ ^and CD8^+ ^T-cell responses but also to affect the functions of a variety of cells, including B, NK, NKT, and dendritic cells [6-10]. Naturally occurring CD4^+^CD25^+ ^regulatory T cells are not suppressive and require T-cell receptor interaction with a specific antigen or polyclonal stimulus to exert suppression. However, once activated, they are able to suppress the proliferation and activation of CD4^+ ^and CD8^+ ^T cells. Because the CD4^+^CD25^+ ^T cell population is heterogeneous, containing activated nonregulatory CD25^+ ^T cells, there have been some contradictory reports about the mechanisms of suppression. However, the suppressive function of CD4^+^CD25^+ ^regulatory T cells is generally considered to be cell contact-dependent and cytokine-independent, although the molecular nature of the contact-dependent interaction has not been fully established. Therefore, for CD4^+^CD25^+ ^regulatory T cells to function efficiently, it is necessary to accumulate them in target organs.

There is also contradictory information about the chemotactic activity of CD4^+^CD25^+ ^regulatory T cells. CD4^+^CD25^+ ^regulatory T cells have been reported to express CCR4, CCR5, CCR7, and CCR8 and to be chemoattracted by their respective ligands, CCL22, CCL4, CCL19, and CCL1 [32-35]. However, Gavin and colleagues [36] showed that CD4^+^CD25^+ ^regulatory T cells were refractory to lymphoid chemokines such as MCP-1, CCL4, CCL19, CCL21, CCL22, and CXCL12. In addition, there are contradictory data about CCR2 expression on CD4^+^CD25^+^CD62^+ ^T cells [35,37]. These discrepancies among the various reports may be because of small variations in the experimental protocol or mouse strains used, but a more important reason may be that distinct cell subsets have unique chemokine responses, and most of the previous studies did not examine these subsets. In the present study, cultured Treg cells had no, or only very low, MCP-1-directed chemotactic activity, whereas CCR2-Treg cells exhibited marked MCP-1-directed chemotaxis.

Pneumonitis and sialadenitis in MRL/lpr mice become noticeable at 10 to 12 weeks of age and progress with age thereafter. Although the mechanism of pneumonitis in MRL/lpr mice is still unknown, disease severity is associated with the degree of mononuclear cell infiltration in the perivascular, peribronchial, and alveolar areas [38]. From our results and other reports, it appears that MCP-1 and IP-10 play an important role in the infiltration of these cells into the lung of MRL/lpr mice [24,26,27]. The development of pneumonitis in MRL/lpr mice is characterized by the accumulation of T cells, especially CD4^-^CD8^-^B220^+ ^and CD4^+ ^T cells [26]. Although the lymphadenopathy in MRL/lpr mice is due to accumulation of CD4^-^CD8^-^B220^+ ^T cells, features of autoimmunity such as production of autoantibodies, arthritis, and sialadenitis depend mainly on CD4^+ ^T cells [39]. Furthermore, activated and clonally expanded CD4^+ ^T cells, which might recognize restricted T-cell epitopes on autoantigens, have been reported to accumulate in the lung of MRL/lpr mice [40]. These findings suggest that CD4^+ ^T cells rather than CD4^-^CD8^-^B220^+ ^T cells play a critical role in the pathogenesis of pneumonitis in MRL/lpr mice. Sialadenitis in MRL/lpr mice is also characterized histopathologically by initial mononuclear cell infiltration into periductular regions, followed by progressive destruction of ductules and parenchyma, resembling the features of Sjögren syndrome. The inflammatory infiltrate consists predominantly of CD4^+ ^T cells. Our studies have shown that MCP-1 and IP-10 are expressed predominantly in infiltrating macrophages/monocytes and ductal epithelium, respectively, beginning at the early stage of sialadenitis in MRL/lpr mice [22]. Therefore, these chemokines seem to be involved in the process of mononuclear cell infiltration.

On the basis of these results, we investigated whether pneumonitis and sialadenitis in MRL/lpr mice could be ameliorated by accumulation of CCR2-expressing CD4^+^CD25^+ ^regulatory T cells in target organs. Adoptive transfer of 5 × 10^5 ^Treg cells showed little effect on the production of autoantibodies and disease progression. In contrast, transfer of CCR2-Treg cells, even in smaller numbers, ameliorated the progression of both pneumonitis and sialadenitis. This was because sufficient numbers of regulatory T cells to suppress the proliferation and activation of autoreactive T cells, especially CD4^+ ^T cells, migrated into the target organs. On the other hand, there was no significant difference in renal damage among control MRL/lpr mice and mice that underwent transfer of Treg cells or CCR2-Treg cells. Two reasons for this can be considered. One is that the number of cells that migrated into the kidney was insufficient to reduce renal damage. The other is that Treg cells may have had little effect on reduction of renal damage given that macrophages rather than T cells play an important role in the development of nephritis in MRL/lpr mice.

Adoptive transfer of naturally occurring or cultured polyclonal CD4^+^CD25^+ ^regulatory T cells has been shown to exert potentially therapeutic effects on autoimmune diseases in many animal models [1,2]. However, the numbers of transferred CD4^+^CD25^+ ^regulatory T cells are small and work better in a local and specific manner since these cells suppress the immune response to tumors and infections. The use of antigen-specific regulatory T cells has been shown to have the most potential for therapy of organ-specific autoimmune diseases in animal models [11-16]. However, in the majority of autoimmune diseases in humans, the organ-specific antigens remain undefined. Therefore, although some problems such as the most effective vector to employ for humans remain unresolved, in the future our method may have useful applications not only for treatment of autoimmune diseases but also for management of transplant rejection and GVHD in humans.

## Conclusion

In the present study, we prepared CCR2-expressing Treg cells and demonstrated their ability to ameliorate pneumonitis and sialadenitis in MRL/lpr mice by accumulating in target organs. In the future, this method may provide a new therapeutic approach for organ-specific autoimmune diseases in which the target antigens remain undefined.

## Abbreviations

CCR2-Treg cell = CD4^+^CD25^+^Foxp3^+ ^CCR2-transfected T cell; ELISA = enzyme-linked immunosorbent assay; FCS = fetal calf serum; Foxp3 = forkhead box p3; GVHD = graft-versus-host disease; HPRT1 = hypoxanthine phosphoribosyl transferase 1; IC = immune complex; IL-2 = interleukin-2; IP-10 = interferon-inducible protein 10; MCP-1 = monocyte chemoattractant protein-1; MoAb = monoclonal antibody; MRL/lpr = MRL/MpJ-*lpr*/*lpr*; NK = natural killer; NKT = natural killer T; PBS = phosphate-buffered saline; PCR = polymerase chain reaction; qPCR = quantitative real-time polymerase chain reaction; Treg cell = CD4^+^CD25^+^Foxp3^+ ^T cell.

## Competing interests

The authors declare that they have no competing interests.

## Authors' contributions

HH conceived of the study, participated in its design and coordination, carried out the experiments and statistical analysis, and drafted the manuscript. AI and MM participated in the design of the study and carried out the experiments. JY contributed to the preparation of retrovirus. TM contributed to the analysis of real-time PCR and evaluation of tissue sections. MY contributed to the review of the manuscript. All authors read and approved the final manuscript.
